# Hints to Locum Tenentes

**Published:** 1909-11-20

**Authors:** 


### The General Practitioner's Column.

[Contributions to this Column are invited, and, if accepted, will be paid. for.]

### HINTS TO LOCUM TENENTES.

Newly-qualified men in the great majority of cases do one of two things—namely, take a voyage as ship surgeon or act as a locum tenens for a few months. In the former there are new scenes, strange peoples and experiences to wipe away all memories of the hard work of the "final." In a locum tenency, however, we have a form of work and experience as useful to the recently qualified man as a house surgeoncy in a hospital, and as essential for his future success as any hospital course. It affords him a chance of seeing different kinds of practices, from the humble, but busy and often lucrative colliery practice, through all grades to the fashionable West End practice.

A few weeks of this kind of work, too, serves to dispel mistaken ideas and conceptions. Cooped up in hospital in the heart of one of the great cities, the student's mind wanders to the green fields of the country, and he dreams of the nice country practice he is going to have, with one day a week across country in pink, and one now and then among the turnips with a gun in September. All this is very well; but it is desirable that he should know the long night journeys in wet weather, the rides, after hunting, on a tired horse, perhaps, to some distant patient whom he guesses won't pay, which form aspects of the reverse side of the picture.

And so too with the man whose thoughts run on colliery practices, with a big list of names on the roll and the regular levy coming to his pocket fortnightly. But first let him sample the long surgery and dispensary hours, and the monotonously regular night calls that will be his lot.

Such then are some of the illusions that will be dispelled. More serious, and yet it is inevitable, are the ideals that will be laid low in the dust. In hospital he has seen every case studied exhaustively and the latest therapeutic knowledge and apparatus employed. In his locum days he realises for the first time that this standard cannot be maintained. The asepsis of the hospitals is now a counsel of perfection. The omnipresent nurse who did so many things for him—and things too which he omitted to learn—is now absent. His instruments, dispensary, and laboratory are all on a more limited, and possibly more primitive, scale. His task is to do the best with what he has, and even in the moment of his disillusionment and disappointment he should hold fast by his ideals and strive after them. This is a very real matter to some men. The writer knows of one man so disappointed with his first experience of practice that he took an asylum post in a colony and left the country a few weeks after graduation, and another who fled to laboratory work.

But the holding of a locum tenency is not only destructive in its influence. It is as has been pointed out a most useful training-ground for practice.

First of all a man learns bookkeeping, a matter of which he is usually profoundly ignorant. As this is matter of great importance, and one in which a little care may make all the difference between a successful balance-sheet at the end of the year or the reverse, it is one to which he cannot devote too much attention.

He will also learn that there is a great gulf fixed between the hospital and private patient, and, let us hope, the rudiments of the art of making and retaining patients. The capacity to win medals and the capacity to attract patients and retain their confidence are very different things, and very often are not found in the same man, so that he who has not shone in his hospital days may take heart and strive to acquire this which is the art of medicine.

He may learn, too, how to stock a dispensary, the useful mixtures, the convenient stock solutions, the most commonly used pills, and the knack, perchance, of dealing with the persistent commercial man who would load him with instruments he will never use. All this and more may be picked up by the man who goes to locum work with his eyes open.

There are other matters, however, worthy of his attention. He should inform himself of his legal position, and his agreement with his principal should be precise and drawn up in writing. He should know for instance, that all fees of whatever nature belong to his principal. No exception can be claimed for inquest fees, for the signing up of lunatics or the giving of an anæsthetic for another practitioner. At the same time he should know what is legally due to him, and insist on having it. Of importance in this connection are the arrangements made for housing and feeding him during his principal's absence. Sometimes he is left in a partially dismantled house with an odd servant to look after him. If he wishes to protest, the time to do it is before the principal leaves. Sometimes, too, sundry little duties are thrown upon him which in no way can be held to be part of his work. The writer knows of a man who was asked, among other things, to wind the numerous clocks, old fashioned and new, which adorned the house, and of which the principal was inordinately proud. This task had to be done at a certain hour on a certain day. He duly performed this unusual task. The solitary servant remaining at home not knowing what he had done, repeated the dose, with results that can be imagined. In all matters relating to money, the payment of accounts and the collection of fees, no doubt should exist in the locum's mind as to what the wishes of his principal are, and he should be ready to give a clear and complete account of all transactions. Care in these matters is always appreciated, on occasions materially. In this connection it may be said that principals are usually extremely generous. They do not expect much, so that the man who is always at hand when wanted, is courteous to patients and agreeable at home, will find his services sought after on other occasions.

Locum work pays well, so that the man who sticks to it, and, as the saying is, works up a connection, may have constant work, and the means, in a few years, to start on his own account.

